# Age-Related Changes in Neurons and Satellite Glial Cells in Mouse Dorsal Root Ganglia

**DOI:** 10.3390/ijms24032677

**Published:** 2023-01-31

**Authors:** Menachem Hanani, David C. Spray, Tian-Ying Huang

**Affiliations:** 1 Laboratory of Experimental Surgery, Hadassah-Hebrew University Medical Center, Mount Scopus, Jerusalem 91240, Israel; 2 Faculty of Medicine, Hebrew University of Jerusalem, Jerusalem 91240, Israel; 3 Department of Neuroscience, Albert Einstein College of Medicine, Bronx, NY 10461, USA

**Keywords:** dorsal root ganglia, neurons, dye coupling, intracellular recording, membrane excitability, aging, pain, gap junctions

## Abstract

The effects of aging on the nervous system are well documented. However, most previous studies on this topic were performed on the central nervous system. The present study was carried out on the dorsal root ganglia (DRGs) of mice, and focused on age-related changes in DRG neurons and satellite glial cells (SGCs). Intracellular electrodes were used for dye injection to examine the gap junction-mediated coupling between neurons and SGCs, and for intracellular electrical recordings from the neurons. Tactile sensitivity was assessed with von Frey hairs. We found that 3-23% of DRG neurons were dye-coupled to SGCs surrounding neighboring neurons in 8–24-month (Mo)-old mice, whereas in young adult (3 Mo) mice, the figure was 0%. The threshold current for firing an action potential in sensory neurons was significantly lower in DRGs from 12 Mo mice compared with those from 3 Mo mice. The percentage of neurons with spontaneous subthreshold membrane potential oscillation was greater by two-fold in 12 Mo mice. The withdrawal threshold was lower by 22% in 12 Mo mice compared with 3 Mo ones. These results show that in the aged mice, a proportion of DRG neurons is coupled to SGCs, and that the membrane excitability of the DRG neurons increases with age. We propose that augmented neuron–SGC communications via gap junctions are caused by low-grade inflammation associated with aging, and this may contribute to pain behavior.

## 1. Introduction

The proportion of aged people in the population is growing steadily worldwide, which poses considerable social and medical challenges. A major health problem associated with aging is chronic pain [1,2,3]. Epidemiological studies showed a higher prevalence of chronic pain in adults over the age of 65, as compared to the general adult population [1]. The treatment options for pain in the aging population are very limited, and the underlying cause for this prevalence is largely unknown.

In recent years, it has become clear that to fully understand and to treat chronic pain, one must consider not only neurons but also glial cells. It is now established that microglia and astrocytes in the central nervous system (CNS) play important roles in pain mechanisms and may serve as therapeutic targets [4,5,6]. There is emerging evidence that in addition to CNS glia, satellite glial cells (SGCs) in sensory ganglia play a major role in nociception [7,8,9,10]. We have shown that gap junction-mediated coupling among SGCs in mouse sensory ganglia increases progressively with age [11] and that augmented glial coupling is correlated with neuronal hyperexcitability and increased tactile sensitivity [12,13]. Similar evidence for the role of gap junctions in nociception was obtained by other investigators [14,15,16]. Whereas coupling among macroglia is observed almost universally [17,18], coupling between neurons and glia appears to be quite rare, and much of the available information is from studies on neuron–astrocyte co-cultures [19,20,21,22], or at early postnatal ages [23,24]. Neuron–SGC coupling in sensory ganglia has been examined in several studies. Calcium imaging in vivo revealed neuron–neuron interactions in mouse dorsal root ganglia (DRGs) that were mediated by gap junctions, and that involved SGCs [25]. This effect was enhanced after peripheral injury or inflammation. This raised the possibility of neuron–SGC coupling, and was verified by dual patch clamp recordings [25]. This idea is supported by an electron microscopic study where the authors reported the presence of neuron–SGC gap junctions in DRGs from mice [26]. In accord with these observations, dual patch clamp recordings provided direct evidence for neuron–SGC electrical coupling in the short-term cultures of mouse trigeminal ganglia [25,27]. Thus, there is emerging evidence for gap junctions between neuron and SGCs, but no information is available on neuron–glia coupling in the aging nervous system. In the present work, we investigated neuron–SGC coupling in DRGs of young and aging mice, and correlated the findings with electrical recordings from the neurons and with behavioral studies. We found that this coupling increases with age, and in parallel, neuronal excitability and tactile sensitivity are augmented.

## 2. Results

### 2.1. Coupling between Neurons and SGCs

**Figure 1:**
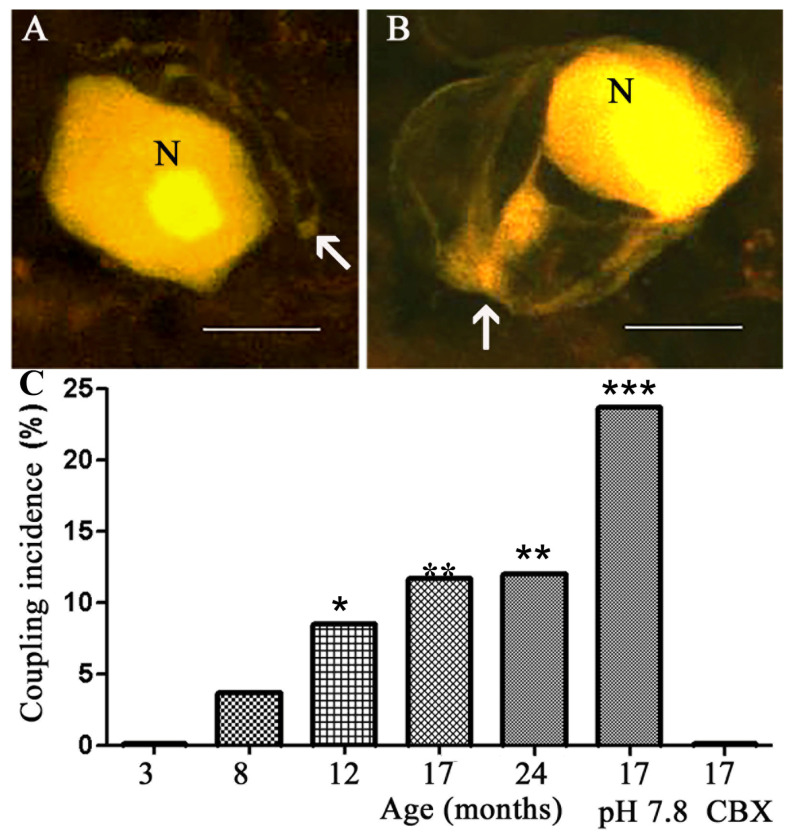
Confocal microscope images of LY-injected neurons in mouse DRGs. (**A**) A neuron (N) with its axon (arrow) in a ganglion from a 3 Mo old mouse is not coupled to SGCs. (**B**) In a ganglion from a 12 Mo mouse, a dye-injected neuron is coupled to SGCs (arrow) surrounding two adjacent neurons. Scale bars, 20 µm. (**C**) Summary of the dye-coupling results. Coupling between neurons and SGCs was observed only in the ganglia from aged mice (8, 12, 17, and 24 Mo). This effect was enhanced when the ganglia were bathed at a slightly basic pH 7.8, and was reduced by the gap junction blocker carbenoxolone (CBX, 50 µM), consistent with gap junction-mediated coupling. The number of cells per data point is 103–164 (72 for CBX results). ANOVA was used for comparison (see Section 4); * *p* < 0.05, ** *p* < 0.01, *** *p* < 0.0001, compared with the control (3 Mo).

The DRGs of young adult (3 Mo) mice served as a reference. The intracellular labeling of neurons in DRGs from these mice showed that none (0/148) of the dye-injected neurons (Figure 1A) were coupled to other cells (either neurons or SGCs), as reported previously [28,29]. In contrast, in DRGs from aged mice (8, 12, 17, and 24 Mo), we found that dye-injected neurons were coupled to SGCs surrounding adjacent neurons in an age-dependent manner (Figure 1). SGCs were identified according to their morphology (nucleated cells surrounding the neuronal somata) and location (very close to the neuronal somata), see [28]. Whether or not the injected neurons were coupled to SGCs surrounding them was difficult to determine, because of the intense staining of the neurons, which would tend to mask the weaker fluorescence of the much smaller SGCs. There was no difference between the results from males and females, and all the data were pooled together. Neuron-to-neuron coupling was not observed at any age.

A low pH reduces the permeability of gap junctions, and a high pH increases it [30]. To test whether the dye coupling was mediated by gap junctions, we incubated the ganglia in a medium with a moderately high extracellular pH—7.8 instead of the normal 7.4, which was shown to increase dye coupling in DRGs [31]. This treatment further increased neuron–SGC dye coupling in ganglia from 17 Mo mice by over two-fold (from 11.7 to 23.7%) (Figure 1C). Conversely, when the neurons were dye-injected in the presence of the gap junction blocker carbenoxolone (CBX, 50 µM), no coupling was observed (Figure 1C). These results indicate that the coupling was mediated by gap junctions. As reported previously for both young and aged mice [11], when SGCs were injected directly, dye passage was detected only to other SGCs, but not to neurons.

### 2.2. Neuronal Excitability Increases with Age

Several studies have shown that augmented cell coupling in DRGs is associated with neuronal hyperexcitability [13,15,25,32], and we asked whether the age-dependent increase in neuron–SGC coupling described above is correlated with changes in the electrical properties of DRG neurons. To test this possibility, we compared the electrophysiological properties of DRG neurons from mice at different ages.

Neurons were classified as A- and C-like type, based on the duration of their action potentials [33]. A-like neurons were defined by a duration <10 ms at baseline, and those with duration >10 ms were classified as C-like cells [34]. Recordings from neurons in DRGs from mice 3 and 12 Mo old revealed the following differences (in both A- and C-like neurons; see Table 1 for the quantitative data): 1. The resting membrane potential (RMP) in the aged mice was lower by about 2 mV. 2. The membrane input resistance (R_in_) was lower in neurons from the 12 Mo mice. 3. The threshold current for evoking an action potential was lower by 20% (A-like type) and 35% (C-like type) in neurons from 12 Mo mice, consistent with greater excitability. 4. The duration of action potentials was longer for neurons from the 12 Mo mice. 5. The number of neurons with spontaneous subthreshold oscillations (SPO) was 2.1-fold greater in neurons from 12 MO mice. The presence of SPO is relevant to neuronal excitability, because the depolarization phase of the oscillations brings the membrane potential closer to the threshold of firing action potentials [35]. Neurons with spontaneous action potential (SPS) were rare in both age groups. The electrophysiological properties of the neurons from 17 and 12 Mo mice were quite similar, see Table 1.

Strikingly, the age-related changes in the neuronal excitability in the 12 Mo mice were completely inhibited by the gap junction blocker CBX (50 µM). In the presence of CBX, the resting membrane potential, the membrane input resistance, and the current threshold for firing an action potential in 12 Mo mice were very similar to those in 3 Mo mice. In the presence of CBX, the number of DRG neurons with spontaneous subthreshold membrane potential oscillations in 12 Mo mice decreased from 38.6% to 17.8% in A-like cells, and from 41.7% to 16.2% in C-like neurons (both *p* < 0.01). CBX did not affect the electrical properties of DRG neurons in 3 Mo mice. This point is relevant, because in addition to blocking gap junctions, CBX can have other effects, and the absence of the changes in control mice indicates that CBX acted mainly on gap junctions.

In summary, intracellular recordings showed that the membrane excitabilities of DRG neurons in 12 and 17 Mo mice were higher than that in 3 Mo mice, and that the age-related changes in the excitability were fully reversed by CBX.

### 2.3. Age-Related Change in Tactile Sensitivity

**Figure 2:**
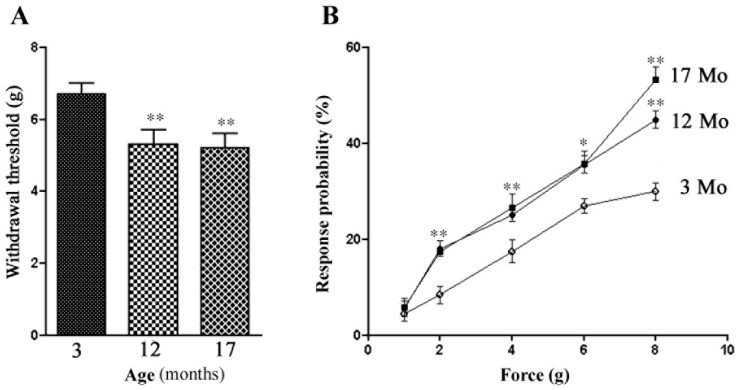
Changes in pain behavior in aged mice. The withdrawal thresholds to the mechanical stimulation of the hind paw skin were assessed with von-Frey hairs. (**A**) Withdrawal thresholds of 12 Mo (*n* = 16) and 17 Mo (*n* = 16) mice are much lower than those of 3 Mo animals (*n* = 20). (**B**) The response probabilities for 12 and 17 Mo mice were also higher than for 3 Mo mice. The data are expressed as mean ± SEM, and the Mann–Whitney test was used for comparison; ** indicates *p* < 0.01.

Changes in the membrane excitability of DRG neurons in the aged animals are expected to influence nociception, and to test this, we used von-Frey hairs to assess the threshold for the withdrawal response to the mechanical stimulation of the hind paws. The withdrawal threshold of aged mice was much lower than for young adult ones, decreasing from 6.8 ± 0.34 g (*n* = 20) in 3 Mo mice to 5.3 ± 0.36 g in 12 Mo mice (*n* = 16, *p* < 0.001 Mann–Whitney test). The response probability was higher in 12 and 17 Mo (Figure 2). These results showed that the aged mice were more sensitive to tactile stimuli than the young adult ones.

## 3. Discussion

The effects of aging on the nervous system have been studied mostly in the central nervous system, and less is known about the influence of age on peripheral sensory neurons and glial cells. Here, we focused on mouse DRGs, and we studied the effects of aging on neuron–SGC coupling and on neuronal electrophysiological properties. We also compared the withdrawal threshold of aged (12 Mo) mice with that of the young adult ones (3 Mo). Neuron–glia coupling by gap junctions was absent in 3 Mo mice, but was observed in the aged mice. Neurons in aged mice displayed hyperexcitability, which was reduced by blocking gap junctions, suggesting a role for neuron–SGC coupling in this effect. The behavioral experiments showed a greater tactile sensitivity in aged mice. Gap junction-mediated coupling among DRG cells was found to augment neuronal activity in pain models in intact mice [25], in agreement with the present results.

There is no general agreement on the topic of pain sensitivity and aging, but most studies support the idea that mechanical sensitivity is greater in the aging population; for reviews, see [1,36]. It was reported [37] that C57B/6 mice showed no change in mechanical sensitivity with age. However, we used Balb/c mice, which may explain the apparent disagreement. Strain dependence in pain behavior is well known; for example, it has been shown that Balb/c mice display much lower withdrawal threshold to von Frey hair stimulation than C57B/6 mice [38].

We showed here that mouse DRG neurons are coupled to SGCs around neighboring neurons in an age-dependent manner (Figure 1). This coupling was blocked by CBX and augmented by a moderate increase in the pH of the medium, indicating that the intercellular dye passage occurred via gap junctions [31]. We have reported previously that SGC–SGC dye coupling and the number of gap junctions increased with age in mouse DRGs [11]. An increase in the number of gap junctions with aging was also found in rabbits [39]. Thus, it appears that aging induces a tendency in DRG neurons and SGCs to form gap junctions. In his respect, aging has similar effects as injury and inflammation, which also increase cell coupling, neuronal excitability, and pain (see below). Various ultrastructural changes have been reported in DRGs of old animals, including a reduction in the number of SGCs and a lowering of both the mitochondrial mass and the mean percentage of cytoplasmic volume occupied by mitochondria [40,41], which may reduce the ATP supply to the cells. It might be speculated that the increased coupling protects in part from these age-related changes, as the gap junctions allow for the passage of ATP and other metabolites, as well as signaling molecules between cells. It should be mentioned that although we present evidence that cell coupling increases with age, and that blocking it reduces neuronal excitability, we did not show that blocking gap junctions can reduce tactile hypersensitivity in the old mice. Such experiments still need to be conducted.

Gap junctions are composed of proteins called connexins (Cxs). The main Cx in SGCs under normal conditions is Cx43 [7]. The present results indicate that gap junctions exist between sensory neurons and SGCs, but the type of the neuronal Cx is not known. It was found that DRG neurons in adult rats contain Cx36, and that the expression of Cx36 RNA was reduced by nerve injury [42]. Thus, it appears that in addition to Cx43, other Cx types are present in the neurons, which can be modified by injury or aging, and this issue still needs to be explored.

When LY was injected directly into SGCs we observed that the dye passed to other SGCs, but not to neurons, in agreement with our previous study on aging mice [11]. It would appear that the coupling between neurons and SGCs is not symmetric; however, we propose a mechanism that is consistent with previous work. In a dual patch clamp recordings study, we showed that SGC–SGC junctional conductance was more than 2-fold greater than for SGC–neuron couples [27]. Thus, it is very likely that the dye passes more rapidly from the injected SGC to other SGCs via the higher conductance pathway, rather than to neurons.

We also found that both A- and C-like neurons in 12 Mo mice showed a lower resting membrane potential, a lower current threshold for firing an action potential, and a higher membrane input resistance than 3 Mo mice. The percentage of neurons with spontaneous subthreshold membrane potential oscillations was also larger in comparison with the young adult mice (Table 1). These findings are consistent with a higher excitability of DRG neurons in the aged mice. The mechanisms underlying these changes are not fully clear, but we propose that the explanation offered previously for pain models may hold in the case of aging. We have suggested that injury leads to augmented intercellular coupling and a greater sensitivity to the pain mediator ATP, acting via purinergic P2 receptors [9,43]. Gap junctions and P2 signaling are major factors enabling the spread of excitation via calcium waves. Currently there is no available information on changes in P2 receptors or in ATP release in old age, but it was found that in the brain, there is an age-related decline in the P2 signaling involving both neurons and astrocytes [44]. Thus, further experiments are needed to test the effect of aging on P2 signaling in sensory ganglia.

The changes in neuronal membrane properties require discussion. We observed that a small but significant depolarization in the neurons of aged mice could increase excitability. On the other hand, neurons from aged animals displayed lower R_in_, which would reduce excitability. The outcome of these opposing effects is difficult to predict, but the observed result is augmented excitability, indicating that the excitatory component is dominant. A similar situation was noted in a model of orofacial pain [34], which indicates that the present results represent a general behavior of sensory neurons under injury and aging.

Neuron–SGC coupling has been observed in the trigeminal ganglia in a systemic inflammation model in mice induced by lipopolysaccharide [27]. In the present work, the mice did not receive any treatment, and apparently, old age has inflammatory-like effects. Indeed, there is strong evidence that aging is associated with low-grade inflammation, which has led to the concept of ‘inflammaging’ [45]. The mechanisms underlying the age-dependent changes reported here are not clear, but it may be proposed that the age-related activation of the immune system leads to the release of proinflammatory agents, which may affect (directly and indirectly) the DRG cells. Such agents may be cytokines [45] or free radicals such as nitric oxide [46]. There is evidence that nitric oxide activates SGCs in DRGs, which leads to neuronal hyperexcitability [47], and it appears that our results are consistent with the inflammaging idea.

## 4. Materials and Methods

### 4.1. Animals and Preparations

Experiments were performed on Balb/c mice of either sex (M:F 1:1), divided into five age groups: 3, 8, 12, 17, and 24 Mo. The experimental protocol was approved by the Institutional Animal Care and Use Committee. The animals were sacrificed via CO_2_ inhalation, and DRGs L4,5 were removed and placed in cold (4 °C) Krebs solution (pH 7.4) containing (mM): 120.9 NaCl, 4.7 KCl, 14.4 NaHCO_3_, 2.5 MgSO_4_, 1.2 NaH_2_PO_4_, 2.5 CaCl_2_, and 11.5 glucose. The DRGs were pinned onto the silicon rubber-covered bottom of a chamber superfused with Krebs solution bubbled with 95% O_2_ and 5% CO_2_ at 23–24 °C for dye injection, or at 32 °C for intracellular recording experiments.

### 4.2. Intracellular Labeling and Recording

Experiments were performed using an upright microscope (Axioskop FS, Zeiss, Jena, Germany) equipped with fluorescent illumination, and a digital camera (Pixera 120e, Pixera Corp., Los Gatos, CA, USA) connected to a PC. In dye injection experiments DRG neurons or SGCs were singly injected with Lucifer yellow (LY, Sigma, 3% in 0.5 M LiCl) from a glass microelectrode connected to a preamplifier (Neuro Data Instrument Corp. model IR 283, New York, NY, USA). The tip resistances of microelectrodes were 80-120 MΩ, and the dye was injected via hyperpolarizing current pulses, 100 ms in duration and 0.5 nA in amplitude at 5 Hz for 3–5 min. During and after dye injections, the LY-labeled cells were imaged with a digital camera. After the experiments, the DRGs were fixed for 20 h at 4 °C in 4% paraformaldehyde in phosphate-buffered saline (PBS, pH 7.4), washed with PBS, and mounted in Gel/mount (Biomeda, Foster, CA, USA). The LY-labeled cells were imaged with a confocal microscope (Biorad, Hercules, CA. USA).

For intracellular recordings, we used sharp glass microelectrodes filled with 2 M KCl, with tip resistances of 80–120 MΩ. Transmembrane currents were passed through the recording electrode using the bridge circuit of a preamplifier (Neuro Data Instrument Corp., NY, model IR 283). The input resistances of the neurons were measured by passing hyperpolarizing currents (0.1 nA and 100 ms) and balancing the bridge. Electrophysiological data were recorded using a Digitizer (DigiData 1200, Axon Instruments, Molecular Devices, San Jose, CA, USA) and AxoScope 9.0 software (Axon Instruments). Neuronal excitability was assessed by measuring the minimal depolarizing current required for eliciting an action potential.

### 4.3. Measurement of Withdrawal Threshold

Von-Frey hairs were used to measure the withdrawal responses to the mechanical stimulation of the plantar skin of the hind paw. Before the behavioral tests, the animals were allowed to become accustomed to the new environment for at least 30 min. The appearance of the following behaviors on the application of a hair was considered as a withdrawal response: (a) sharp and quick leg withdrawal; (b) immediate jumping. Hairs calibrated for forces of 1.0, 2.0, 4.0, 6.0, and 8.0 g were applied 10 times each, in ascending order of force. Both pain threshold and response probability were examined. The hair was applied for 1–2 s at 5–10 s intervals. Care was taken not to stimulate the same point in succession. The withdrawal threshold was defined as the minimum force eliciting two subsequent withdrawal responses [13].

### 4.4. Statistical Analysis

All average values are expressed as mean ± SEM. A Mann–Whitney test was used for the comparisons. Dye coupling data were pooled for each of the experiments; this was performed because in different dye-coupling experiments, different numbers of cells were injected, and relatively small numbers of cells were injected per experiment. When an LY-injected neuron was found to be dye-coupled, it was marked as 100, and when it was not coupled, as 0. These data were analyzed using a one-way ANOVA with Tukey’s multiple comparison. *p* < 0.05 was considered as statistically significant.

## Figures and Tables

**Table 1:** Electrophysiological properties of neurons in DRGs of 3, 12, and 17 Mo mice.

**A-like Type Neurons**					
**Age (months)**	**3**	**3+CBX**	**12**	**12+CBX**	**17**
*n*	63	34	59	45	47
RMP (mV)	49.9 ± 1.1	50.1 ± 1.3	44.7 ± 0.9 *	49.7 ± 1.1	46.5 ± 0.8 *
APA (mV)	55.8 ± 1.6	58.1 ± 1.7	54.5 ± 1.7	59.1 ± 1.4	57.6 ± 1.5
APD (ms)	5.8 ± 0.2	6.1 ± 0.3	6.5 ± 0.2 *	6.1 ± 0.3	6.3 ± 0.3
Threshold current (nA)	0.42 ± 0.03	0.43 ± 0.04	0.34 ± 0.03 *	0.45 ± 0.03	0.30 ± 0.03 *
Ri (MΩ)	42.1 ± 0.7	43.2 ± 1.1	46.2 ± 0.8 *	41.1 ± 0.8	46.0 ± 0.8 *
SPS	2 (3.2%)	1(2.9%)	2 (3.4%)	0	3 (6.4%)
SPO	12 (19%)	6 (17.6%)	22 (38.6%) *	8 (17.8%)	18 (38.3%) *
**C-like Type Neurons**					
*n*	38	28	23	37	35
RMP (mV)	51.7 ± 1.4	52.0 ± 1.6	47.0 ± 1.6 *	53.1 ± 1.2	48.4 ± 0.9 *
APA (mV)	65.1 ± 1.8	64.6 ± 2.3	60.2 ± 2.5	66.2 ± 1.6	61.7 ± 1.4
APD (ms)	11.7 ± 0.4	12.1 ± 0.4	13.2 ± 0.6 *	13.1 ± 0.4*	12.9 ± 0.4 *
Threshold current (nA)	0.38 ± 0.04	0.39 ± 0.04	0.25 ± 0.04 **	0.36 ± 0.02	0.25 ± 0.03 **
Ri (MΩ)	46.6 ± 0.09	45.9 ± 1.1	49.9 ± 1.2 *	43.5 ± 1.0	49.8 ± 1.1 *
SPS	1 (2.6%)	0	1 (4.3%)	0	3 (8.6%)
SPO	7 (18.4%)	5 (17.8%)	10 (41.7%) *	6 (16.2%)	14 (40%) *

Data are presented as mean ± SEM. * *p* < 0.05, ** *p* < 0.01, as compared with the control (3 Mo), Mann–Whitney test. CBX indicates that CBX (50 µM) was added in the bathing medium of the DRGs. RMP, resting membrane potential; APA, action potential amplitude; APD, action potential duration (at resting potential); Ri, membrane input resistance; SPS, spontaneous spikes; SPO, spontaneous oscillations.

## Data Availability

Available on request.
